# SLEEP DISORDERS AND THEIR IMPACTS ON OLDER ADULT SPOUSAL RELATIONSHIP

**DOI:** 10.1093/geroni/igae098.0209

**Published:** 2024-12-31

**Authors:** Sophia Geisser, Ashley Fromenthal, Erick Carranza, Michelle Hilgeman, A Lynn Snow

**Affiliations:** University of Alabama, Tuscaloosa, Alabama, United States; University of Alabama, Tuscaloosa, Alabama, United States; University of Alabama, Tuscaloosa, Alabama, United States; Tuscaloosa VA Medical Center, Tuscaloosa, Alabama, United States; University of Alabama, Tuscaloosa, Alabama, United States

## Abstract

Aging is associated with biological and psychosocial impairments that affect individuals’ sleep and quality of life. Approximately half of older adults suffer from a sleep disorder. Daytime consequences such as fatigue, excessive sleepiness, irritability, mood swings, and/or cognitive difficulties may be present with sleep disorder diagnoses, impacting interpersonal relationships. There is limited research on associations between sleep disorder and spousal relationship quality in older adults. The need to expand the field of research in sleep disorders is urgent, as sleep disorder diagnoses are expected to rise with the growing older adult population. The objective of this presentation is to describe a conceptual model of sleep disorders and relationship quality informed by the Meikirch model of health: sleep disorders represent biologically given potential, relationship quality represents personally acquired potential, and the COVID-19 pandemic represents the environmental determinants of health. Applying this theoretical underpinning, the Health and Retirement Study data from 2020 was analyzed via simple linear regression analyses to assess the relationships between sleep disorders and perceived life satisfaction in spousal relationships. Results show that having a sleep disorder significantly predicted life satisfaction: F(1,4647) = 40.49, p <.001. (b =.09, S.E. =.02, t = 6.36, p <.001, 95%CI[.06,.13]); and having a sleep disorder significantly predicted perceived problems in spousal relationships: F(1,4560) = 15.49, p <.001 (b = -.12, S.E. =.03, t = -3.94, p <.001, 95%CI[-.17,.06]). Understanding sleep-related associations may inform interventions to promote well-being and life satisfaction for older adult couples.

